# Triglyceride-glucose index as predictor of gestational diabetes mellitus in early pregnancy: a prospective cohort study

**DOI:** 10.3389/fendo.2026.1875641

**Published:** 2026-06-29

**Authors:** Ge Huang, Meng Su, Cheng Li, Hongzhuan Tan, Jing Deng, Mengshi Chen

**Affiliations:** 1 Department of Epidemiology and Health Statistics, Xiangya School of Public Health, Central South University, Changsha, China; 2 Hunan Provincial Key Laboratory of Clinical Epidemiology, Xiangya School of Public Health, Central South University, Changsha, China

**Keywords:** changes during pregnancy, gestational diabetes, prospective cohort study, risk of onset, TyG index

## Abstract

**Background/objectives:**

Gestational diabetes mellitus (GDM) poses a serious threat to the health of both mothers and infants, and currently, methods for early and accurate prediction remain limited. This study aims to investigate the association between TyG levels in early pregnancy, TyG levels in mid-pregnancy, and their dynamic differences with the risk of developing gestational diabetes (GDM), thereby providing clinical guidance for the early prevention and control of GDM during pregnancy and the management of high-risk populations.

**Methods:**

A prospective cohort study design was employed, enrolling 870 pregnant women. Variables were screened using univariate Cox regression, followed by the construction of a multivariate Cox proportional hazards regression model to analyze the independent association between the percentage increase in TyG levels and the change in TyG levels at different time points and the incidence of GDM.

**Results:**

Among the 870 study participants, 119 developed GDM, resulting in an incidence rate of 13.68%. Multivariate Cox regression analysis showed that, after adjusting for confounding factors such as age, BMI, and family history of diabetes, elevated TyG levels in early pregnancy, elevated TyG levels in mid-pregnancy, and a greater percentage increase in the difference between TyG levels in early and mid-pregnancy were all independent risk factors for GDM, with elevated TyG levels in mid-pregnancy posing the highest risk.

**Conclusion:**

Elevated TyG indices during the first and second trimesters, as well as a progressive increase in TyG levels throughout pregnancy, are all independent risk factors for the development of gestational diabetes mellitus (GDM). These factors have certain reference value for evaluating GDM risk and provide a basis for the early prevention and management of gestational diabetes.

## 1 Introduction

Gestational diabetes mellitus (GDM) refers to impaired glucose tolerance of varying degrees that first appears or develops during pregnancy (1); it is considered a distinct category separate from Type 1 diabetes mellitus (T1DM) and Type 2 diabetes mellitus (T2DM). In 2013, the International Diabetes Federation (IDF) conducted a comprehensive statistical analysis of epidemiological data from 47 studies across 34 countries and regions worldwide to estimate the prevalence of GDM among pregnant women aged 20–49 (2), ultimately reporting an overall prevalence of 14.2%; In China, the prevalence is approximately 5%–10% (3), which remains relatively low on a global scale. However, due to China’s large population, the number of women affected by GDM in 2013 still exceeded 1 million, ranking second worldwide. In addition to the data published by the IDF in 2013, Chinese researchers have also conducted large-scale epidemiological surveys on the incidence of GDM in China, revealing a trend of increasing prevalence year by year (4–9). Although this increase may be partly attributed to the lowering of diagnostic thresholds under new criteria, the actual rise cannot be ignored.

Some scholars believe that the prevalence of GDM is one of the key factors contributing to the global spread of T2DM and metabolic syndrome (MS) (10, 11). GDM poses a serious threat to maternal and fetal health, increasing the risk of maternal pregnancy complications such as gestational hypertension, polyhydramnios, premature rupture of membranes, miscarriage, dystocia, and birth canal injuries, as well as increasing the risk of preterm birth, macrosomia, congenital malformations, and neonatal hyperbilirubinemia in the fetus. This is primarily because glucose can diffuse from the mother to the fetus, but maternal insulin does not enter the fetus at the same rate. Consequently, maternal GDM exposes the fetus to higher glucose concentrations than normal, forcing the fetus to increase its own insulin production, leading to fetal overgrowth and the development of a macrosomic infant. During delivery, a macrosomic infant not only increases risks to itself, such as shoulder dystocia and neonatal asphyxia (12, 13), but also increases the risk of maternal injury. In addition, fetuses exposed to a high-glucose environment may develop other complications after birth, including neonatal respiratory distress syndrome, cardiomyopathy, hypoglycemia, hypocalcemia, hypomagnesemia, polycythemia, and hyperviscosity syndrome (14).

In recent years, many medical organizations have successively issued new guidelines for the diagnosis and management of gestational hyperglycemia. These guidelines generally recommend screening for GDM between 24 and 28 weeks of gestation (mid-to-late pregnancy), and interventions to reduce maternal hyperglycemia starting from the time of diagnosis have been shown to significantly improve short-term pregnancy outcomes (15, 16). However, a growing body of evidence suggests that current approaches may fail to mitigate the long-term adverse effects resulting from prolonged fetal exposure to high blood glucose levels *in utero*. For example, a study by Sovio et al. (17) demonstrated that fetal growth acceleration occurs as early as 20 weeks of gestation, preceding the diagnosis of GDM. Concurrently, a study by Logan et al. (18) confirmed that, despite good maternal glycemic control, offspring of mothers with GDM exhibit marked obesity in early infancy, further supporting the importance of early detection and treatment of GDM. There is also ample evidence indicating that lifestyle interventions initiated before pregnancy or in early pregnancy (gestational age <20 weeks) in high-risk women can effectively prevent the onset of GDM and its complications (19–22). However, a major issue in current research lies in the fact that different researchers rely on varying diagnostic criteria for GDM, propose predictive equations with different risk factors, and obtain predictive equations with significantly differing sensitivity and specificity. Consequently, the early diagnosis of GDM still requires further exploration (23, 24). Since GDM is also influenced by genetic susceptibility, the incidence of GDM varies across different ethnic groups; consequently, the early diagnosis of GDM should differ among these groups. Currently, early diagnosis of GDM in China remains relatively underdeveloped, making research on early prediction of GDM in the Chinese population essential.

Triglycerides - The Triglyceride-to-Glucose Ratio (TyG) is a simple indicator reflecting disorders of glucose and lipid metabolism and insulin resistance. The TyG ratio is a convenient, stable, and clinically applicable measure for assessing insulin resistance; it can be calculated using only fasting blood glucose and triglyceride levels, without the need for insulin testing, and holds significant value in the screening and risk prediction of metabolic-related diseases (25). In recent years, multiple studies have confirmed that an elevated TyG index is closely associated with the risk of developing type 2 diabetes, metabolic syndrome, and cardiovascular disease, and can serve as an early warning indicator of abnormal glucose and lipid metabolism (26). A retrospective cohort study by Zhang Jie (27) et al. confirmed that the TyG index is independently and positively associated with the risk of developing GDM; the triglyceride-to-glucose ratio (TyG) and its derived composite indices demonstrate significant value in the risk assessment and mechanistic investigation of gestational diabetes (GDM). During pregnancy, the exacerbation of insulin resistance is the core pathophysiological mechanism underlying the development of gestational diabetes (25, 28). Yanan Duan (29) and colleagues, in a large-scale study of a U.S. population, further found that TyG mediates the association between a history of GDM and elevated levels of systemic chronic inflammation, revealing that metabolic dysfunction is a key intermediary pathway through which GDM leads to long-term inflammatory damage.Therefore, the TyG index holds promise as a predictor of the risk of developing gestational diabetes, offering a simple and feasible tool for the early identification of abnormal glucose metabolism during pregnancy.In addition, the composite indicator TyG-BMI, which incorporates body mass index, also shows great promise for clinical application. Xiaomin Liang et al. (30) reported in a prospective cohort study in South Korea that elevated TyG-BMI levels in early pregnancy significantly increase the risk of GDM. This indicator demonstrates excellent diagnostic performance and can serve as an effective tool for the early screening of GDM.

## 2 Materials and methods

### 2.1 Data sources and study population

The study began recruiting pregnant women in the first trimester to establish a cohort at the Hunan Provincial Maternal and Child Health Hospital in late October 2016. The study protocol was approved by the Medical Ethics Committee of Hunan Provincial Maternal and Child Health Hospital (Approval No. EC201624). Prior to the survey, the study details were fully explained to the participants, and all participants signed informed consent forms. The study was conducted in full compliance with the Declaration of Helsinki. Inclusion criteria for study participants were as follows: singleton, naturally conceived pregnancy; intention to undergo all prenatal care and delivery at this hospital; no history of chronic metabolic or cardiovascular diseases (such as diabetes, hypertension, thyroid disorders, or cardiovascular/cerebrovascular diseases) prior to pregnancy; no history of long-term medication use affecting glucose or lipid metabolism; no acute infections in the past 2 weeks; and no use of antibiotics during pregnancy; pregnant women in the first trimester (10–13^+6^ weeks) planning to undergo prenatal care and delivery at Hunan Provincial Maternal and Child Health Hospital. This stage is the standard time for the first systematic prenatal examination, facilitating uniform clinical procedures and data collection; at the same time, changes in metabolic indicators at this stage can reflect insulin resistance at an earlier stage, providing a basis for the early risk assessment of gestational diabetes. Given that physiological changes at different gestational weeks may affect TyG index levels, this study uniformly collected fasting venous blood samples at the aforementioned time point to standardize the testing time, minimize the impact of gestational week.Clear exclusion criteria were also established, including: pre-pregnancy diagnosis of diabetes or impaired glucose tolerance; multiple gestation or ectopic pregnancy; severe liver or kidney dysfunction or malignant tumors; serious complications during pregnancy such as preterm rupture of membranes or placental abruption; and subjects who were lost to follow-up or had significant data gaps during the study period. Following the collection of baseline data in early pregnancy, the study will conduct prospective follow-up for all participants until 24–28 weeks of gestation, and the incidence of gestational diabetes mellitus (GDM) will be determined based on the results of the 75-gram oral glucose tolerance test (OGTT). Diagnostic criteria were based on the Chinese Guidelines for Maternal-Fetal Co-management of Gestational Diabetes (2024 Edition): In China, the 75-g oral glucose tolerance test (OGTT) is used to diagnose GDM. The diagnostic criteria are as follows: 5.1 mmol/L ≤ fasting plasma glucose (FPG) <7.00 mmol/L, 1-hour OGTT glucose ≥ 10.0 mmol/L, and 2-hour OGTT glucose between 8.5 mmol/L and 11.1 mmol/L. A diagnosis of GDM is made if glucose levels at any of these time points meet the above criteria.

### 2.2 Data collection

After screening, pregnant women in the first trimester who met the criteria were first briefed on the study and asked for their consent. Only those who were willing to actively cooperate with all study procedures were finally enrolled after signing an informed consent form. Subsequently, each participant was assigned a unique identification number on-site in sequential order, and this number was used to identify all subsequent data, including questionnaires and biological samples. Basic demographic information was collected via a questionnaire at the time of enrollment. The questionnaire is provided in Appendix A. Physical examination and laboratory test results were obtained strictly in accordance with established clinical testing standards, All blood tests in this study were conducted using standardized operating procedures under fasting conditions to minimize random errors associated with individual measurements. The information collected is as follows:

Demographic data: Subject’s age, educational level, household income, marital status, parity, number of previous births, history of adverse pregnancy outcomes (including spontaneous abortion, preterm birth, and fetal malformations), and family history of diabetes.
Physical Examination Parameters: Measurement of participants’ height, pre-pregnancy weight, and systolic and diastolic blood pressure in early pregnancy.
Laboratory test indicators: Fasting blood glucose, triglycerides, high-density lipoprotein cholesterol (HDL-C), low-density lipoprotein cholesterol (LDL-C), and other relevant indicators.
Lifestyle and medical history: Physical activity (exercising ≥3 times per week, at least 30 minutes per session), sedentary behavior, history of secondhand smoke exposure, history of alcohol consumption before pregnancy, history of smoking, and history of gynecological conditions such as polycystic ovary syndrome (PCOS) and endometriomas.

Based on the data and laboratory parameters collected above, this study further calculated relevant composite variables. The formula for TyG is as follows: TyG = ln [fasting blood glucose (mg/dL) × triglycerides (mg/dL)/2](In this study, the laboratory test results for fasting blood glucose and triglycerides were reported in mmol/L, in accordance with domestic clinical standards. For the calculation of the TyG index, all values were uniformly converted to mg/dL.), with fasting blood glucose and triglyceride data derived from early pregnancy fasting venous blood test results.

### 2.3 Statistical analysis

Statistical analysis was performed using IBM SPSS Statistics 27 software, with a two-sided significance level of α = 0.05; a P-value of <0.05 was considered statistically significant. The overall missing data rate for the study variables was low, with 5.0% missing for waist circumference, 4.8% for diastolic blood pressure, 4.5% for the TyG index in the second trimester, 4.0% for the TyG index in the first trimester, 3.5% for HDL, 3.2% for LDL, and 5.2% for the DII index; The missing rates for questionnaire-based variables such as age, educational level, adverse obstetric history, and family history were all below 2%, while the missing rates for the remaining indicators did not exceed 3%.To ensure the integrity of the data and the reliability of the statistical results, missing data were imputed using the Multiple Imputation by Chains (MICE) method. A total of five imputed datasets were constructed, and the final statistical analysis was based on the merged dataset. Study participants were grouped according to the third quartile of the TyG index in early pregnancy. Continuous variables were described as mean ± standard deviation, and comparisons among groups were performed using one-way analysis of variance (ANOVA). Categorical variables were presented as frequency (proportion), and comparisons between groups were performed using the χ² test. A univariate Cox proportional hazards regression model was used to identify risk factors associated with GDM. Variables showing statistically significant differences in the univariate analysis were included in a multivariate Cox regression model. Both an unadjusted model and an adjusted model (adjusted for age, family history of diabetes, pre-pregnancy BMI, adverse obstetric history, blood lipid levels, and early pregnancy blood glucose) were constructed to investigate the independent association between the TyG index and the risk of GDM.

## 3 Results

This cohort study included a total of 872 participants; 870 participants with complete data were included in the analysis, and the sample was sufficient to meet the requirements for analyzing most suspected risk factors. Among the 870 participants, 119 developed GDM, resulting in a GDM incidence rate of 13.68%.

### 3.1 Analysis of baseline characteristics of the study population and univariate analysis of risk factors for gestational diabetes mellitus

Continuous variables were divided into three or four groups using the tertile or quartile method based on their distribution characteristics and converted into ordinal categorical variables; categorical variables were included in the analysis directly in their original categories. Univariate Cox proportional hazards regression models were used to analyze the association between each factor and the incidence of GDM. Univariate Cox regression analysis revealed that age, adverse obstetric history, family history of diabetes, pre-pregnancy BMI, first-trimester blood glucose, first-trimester waist circumference, high-density lipoprotein cholesterol (HDL), low-density lipoprotein cholesterol (LDL), and first- and second-trimester triglycerides-(TyG) in the first and second trimesters were associated with the risk of GDM (P < 0.05); secondhand smoke, physical inactivity, exercise, educational level, polycystic ovary syndrome, and the Dietary Inflammation Index (DII) were not statistically associated with GDM (P > 0.05).

The incidence of GDM in the first-trimester TyG tertiles was 10.2%, 12.5%, and 18.5%, respectively; compared with the low TyG group, the risk of GDM was significantly higher in the high TyG group (HR = 1.885, 95% CI: 1.203, 2.955). The incidence rates of GDM in the three TyG tertiles during the second trimester were 6.6%, 10.8%, and 23.9%, respectively, showing a clear upward trend; Cox regression analysis indicated that the risk of GDM in the high TyG group was 3.996 times that of the low TyG group; see **Table 1** for details.

**Table 1:** Results of univariate Cox regression analysis of factors associated with GDM.

Variable	Number of people in the queue	Number of GDM cases	GDM incidence (%)	HR (95% CI)
Age				
≤35	789	103	13.0	1.000
>35	81	16	20.0	1.578 (0.932, 2.671)
history of adverse pregnancy outcomes				
no	510	51	0.1	1.000
yes	360	68	0.19	1.993 (1.386, 2.865)
Secondhand smoke				
no	698	93	0.13	1.000
yes	172	26	0.15	1.136 (0.736, 1.755)
Prolonged sitting				
no	290	42	0.14	1.000
yes	580	77	0.13	0.911 (0.626, 1.327)
Family History				
no	783	102	0.13	1.000
yes	87	17	0.20	1.536 (0.919, 2.567)
Pre-pregnancy BMI				
Normal or underweight	739	77	0.10	1.000
Overweight	112	35	0.31	2.504(1.542, 4.065)
Obese	19	7	0.37	5.164(2.504, 10.650)
Sports				
no	627	79	0.13	1.000
yes	243	40	0.16	1.310 (0.896, 1.917)
Level of education				
Junior High School	21	3	0.14	1.000
Secondary vocational education	99	13	0.13	0.871 (0.248, 3.058)
Junior college	236	42	0.18	1.212 (0.376, 3.911)
Bachelor	414	49	0.12	0.790 (0.246, 2.536)
Master/Doctor	100	12	0.12	0.796 (0.225, 2.820)
Polycystic ovary syndrome				
no	809	107	0.13	1.000
yes	61	12	0.20	1.537 (0.847, 2.792)
Blood sugar levels in early pregnancy				
≤4.40	229	15	6.55	1.000
4.41-4.60	211	21	9.95	1.519 (0.783, 2.947)
4.61-4.90	239	30	12.55	1.916 (1.031, 3.562)
≥4.91	191	53	27.75	4.236 (2.388, 7.515)
Waist circumference in early pregnancy				
≤73.00	250	20	8.00	1.000
73.01-78.00	230	25	10.87	1.359 (0.755, 2.446)
78.01-82.00	182	23	12.64	1.580 (0.868, 2.876)
≥82.01	208	51	24.52	3.065 (1.827, 5.140)
HDL				
≤1.66	219	49	22.37	1.000
1.67-1.92	220	27	12.27	0.549 (0.343, 0.877)
1.93-2.21	215	20	9.30	0.416 (0.247, 0.699)
≥2.22	216	23	10.65	0.476 (0.290, 0.781)
LDL				
≤2.00	231	26	11.26	1.000
2.01-2.40	225	24	10.67	0.948 (0.544, 1.651)
2.41-2.88	198	28	14.14	1.256 (0.737, 2.143)
≥2.89	216	41	18.98	1.686 (1.032, 2.757)
Early pregnancy TyG				
≤8.36	294	30	10.2	1.000
8.37-8.66	295	37	12.5	1.232 (0.761, 1.994)
≥8.67	281	52	18.5	1.885 (1.203, 2.955)
Second trimester TyG				
≤8.90	290	19	6.6	1.000
8.91-9.24	296	32	10.8	1.661 (0.941, 2.930)
≥9.25	284	68	23.9	3.996 (2.403, 6.647)
DII				
≤-1.13	218	30	13.76	1.000
-1.12-0.31	217	31	14.29	1.054 (0.638, 1.741)
0.32-1.94	218	26	11.93	0.875 (0.517, 1.479)
≥1.95	217	32	14.75	1.083 (0.658, 1.783)
TyG percentage increase (%)				
≤6.4	435	39	8.97	1.000
>6.4	435	80	18.39	2.180 (1.486, 3.197)

### 3.2 Multifactor analysis of risk factors for GDM

To rule out the influence of potential confounding factors and investigate the independent effects of the first-trimester TyG index, the second-trimester TyG index, and the percentage increase in their difference on the incidence of GDM, we used the presence or absence of GDM as the dependent variable. We included variables that showed statistically significant differences in the univariate analysis—first-trimester BMI, adverse obstetric history, HDL, LDL, first-trimester blood glucose, age at delivery, and family history as independent variables in a multivariable Cox model. We established Model 1 (unadjusted) and Model 2 (adjusted for confounding factors). Cox regression analysis revealed that, after adjusting for confounding factors, the high TyG group in early pregnancy (≥8.67) remained an independent risk factor for GDM (HR = 1.782, 95% CI: 1.132, 2.804, P = 0.013). The risk of GDM in the high TyG group (≥9.25) during the second trimester was significantly higher than that in the low TyG group (HR = 3.301, 95% CI: 1.967, 5.539, P < 0.001); An increased percentage change in TyG from early to mid-pregnancy was also independently associated with an elevated risk of GDM (HR = 2.024, 95% CI: 1.399, 2.927, P = 0.001). See **Table 2** for details.

**Table 2:** Results of the multivariate Cox regression analysis of factors influencing GDM.

Variable	No	Yes	Model1 HR (95%CI)	P-value	Model2 HR (95% CI)	P-value
Early pregnancy TyG						
≤8.36	264	30	—	1.000	—	1.000
8.37-8.66	258	37	1.232 (0.761, 1.994)	0.396	1.191 (0.735, 1.930)	0.478
≥8.67	229	52	1.885 (1.203, 2.955)	0.006	1.782 (1.132, 2.804)	0.013
Second trimester TyG						
≤8.90	271	19	—	1.000	—	1.000
8.91-9.24	264	32	1.661 (0.941, 2.930)	0.080	1.643 (0.930, 2.902)	0.087
≥9.25	216	68	3.996 (2.403, 6.647)	0.001	3.301(1.967, 5.539)	0.001
TyG percentage increase (%)						
≤6.4	396	39				
>6.4	355	80	2.180 (1.486, 3.197)	0.001	2.564 (1.734, 3.790)	0.001

TyG Percentage Increase = (Second Trimester TyG − Early Pregnancy TyG)/Early Pregnancy TyG× 100%. This represents the relative change in the TyG index from the first trimester to the second trimester; a positive value indicates that TyG has increased compared to the first trimester, while a negative value indicates that TyG has decreased compared to the first trimester.Percentage changes are used solely for the purpose of standardizing metric presentation, visualizing trends, and making cross-group comparisons; they should not be interpreted as percentages in the strict mathematical sense.

Model 1: Unadjusted model.

Model 2: Adjusted for age at delivery, family history, first-trimester BMI, adverse obstetric history, HDL, LDL, and first-trimester blood glucose.

### 3.3 ROC curve analysis of the TyG index for predicting gestational diabetes

Three predictive models were constructed: one combining the percentage change in TyG with BMI, one combining the first-trimester TyG index with BMI, and one combining the second-trimester TyG index with BMI. ROC curves were plotted to evaluate the predictive value of each indicator for gestational diabetes. The results showed that the area under the curve (AUC) for all three models was greater than 0.5, with statistically significant differences (P < 0.001), indicating that all models can play a certain role in predicting gestational diabetes.

The combination of the first-trimester TyG index and BMI demonstrated the highest specificity, reaching 82.1%, which effectively reduces misdiagnosis; its AUC was 0.683, with a 95% confidence interval of 0.630–0.736. The combination of the TyG index and BMI showed the best predictive performance in the second trimester, with an AUC of 0.731 (95% confidence interval: 0.684–0.779), an optimal cutoff value of 9.85, corresponding sensitivity of 78.5%, specificity of 76.2%, and a Youden index of 0.547. The AUC for the percentage change in TyG was 0.684 (95% confidence interval: 0.631–0.736), indicating relatively weak discriminatory ability; it can serve as an auxiliary clinical assessment indicator.

## 4 Discussion

This study employed a cohort study design, tracking a cohort of 870 pregnant women, among whom 119 developed GDM, resulting in a GDM incidence rate of 13.68%. Based on these findings, we explored the independent association between the TyG index and its percentage change at different stages of pregnancy and the risk of GDM. As a cohort study was employed, recall bias and potential confounding from temporal causality were avoided, ensuring the reliability of the results.

### 4.1 The association between TyG and the risk of GDM in early pregnancy and its clinical implications

After adjusting for confounding factors using multivariate Cox regression, the group with high TyG levels in early pregnancy (≥8.67) still showed a significantly increased risk of GDM (HR = 1.782, 95% CI: 1.132, 2.804, P = 0.013), suggesting that elevated TyG levels in early pregnancy are an independent risk factor for GDM.

Early pregnancy is a critical stage for maternal metabolic reprogramming and placental development. Elevated TyG levels at this stage primarily reflect pre-existing, latent insulin resistance and dysregulation of glucose and lipid metabolism. Under the combined effects of pregnancy-induced insulin-antagonistic hormones, the body’s ability to compensate for glucose metabolism declines, leading to the progression of GDM. A prospective cohort study by Duoyanbei et al. (31). conducted a prospective cohort study of Chinese pregnant women, confirming that TyG in early pregnancy exhibits a dose-response relationship with GDM and can serve as a stable early metabolic biomarker. Li et al. (32). found that the risk of GDM in the group with high TyG in early pregnancy was 3.14 times higher (OR: 3.14; 95% CI: 2.55–3.85) compared to the group with lower risk, consistent with the trend observed in this study. Song et al. (33). noted that logistic regression analysis showed that, after adjusting for covariates, a 1-unit increase in the TyG index was associated with a 2.21-fold increase in the risk of GDM, and this result differed significantly across all quartiles. Gao Sheng et al. (34). Confirmed, based on a large-scale cohort of pregnant women, that elevated TyG in early pregnancy is an independent risk factor for GDM. This finding corroborates the results of this study, which identified both early and mid-pregnancy TyG as independent risk factors for GDM, thereby providing external evidence supporting the conclusions of this study in the Chinese population. Ge Xuhong et al. (35). demonstrated in a general adult population that TyG and TyG-BMI can reliably predict the risk of diabetes, which is highly consistent with the conclusion of this study that TyG combined with BMI predicts GDM in pregnancy, suggesting that the combination of TyG and BMI is a high-quality metabolic predictor applicable across populations.

In clinical practice, the TyG index in early pregnancy can be calculated using only routine fasting blood glucose and triglyceride measurements. This approach is cost-effective and highly accessible, making it suitable for developing an early risk stratification tool. During the first prenatal visit in early pregnancy, fasting blood glucose and triglyceride levels should be measured simultaneously, the TyG index calculated, and combined with pre-pregnancy BMI. The optimal cutoff values derived from this study should then be used to stratify pregnant women by risk. Pregnant women with a TyG index above the cutoff value are identified as high-risk for GDM. Subsequently, they can be managed with targeted interventions, such as increased frequency of glucose monitoring and adjustments to dietary and exercise guidance, to achieve early identification of GDM and individualized intervention.

### 4.2 The association between TyG and the risk of GDM in mid-pregnancy and its clinical implications

This study found that the association between TyG in the second trimester and GDM was significantly stronger than that in the first trimester. In univariate analysis, the risk of GDM in the group with high TyG (≥9.25) in the second trimester was 3.996 times higher than that in the low group; After adjusting for confounding factors, the HR was 3.301 (95% CI: 1.967, 5.539; P < 0.001), and it remained the strongest independent risk factor.

**Figure 1:**
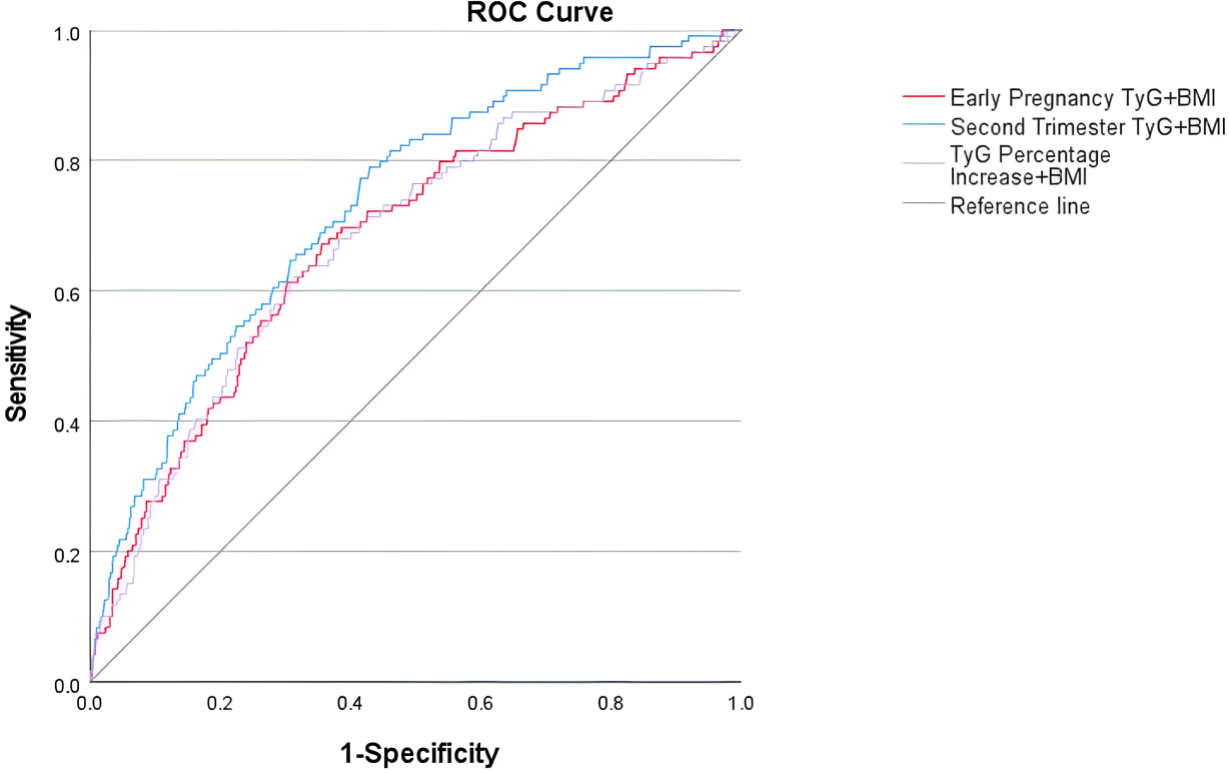
ROC curve for predicting GDM using the TyG index and combined pre-pregnancy BMI.

Maternal physiological insulin resistance peaks during the second trimester, which is the critical window for the onset of GDM; TyG can accurately reflect the degree of glucose and lipid metabolism disorders and insulin resistance (as shown in **Figure 1** and **Table 3**). The ROC analysis in this study showed that the AUC for predicting GDM using the mid-pregnancy TyG index alone was 0.690; predictive performance was further enhanced when combined with pre-pregnancy BMI, confirming that the mid-pregnancy TyG index possesses moderate predictive value for GDM. A related domestic study by Wu Lulu et al. (36). similarly noted that the mid-pregnancy TyG index is an independent risk factor for GDM, with clear predictive value for the condition; combining it with relevant anthropometric indicators can effectively improve overall screening accuracy. The risk association conclusions and predictive performance results of this study complement and support the findings of that study.

**Table 3:** ROC analysis results for the TyG index combined with BMI in predicting GDM.

Measure	Cutoff	AUC (95% CI)	P	Sensitivity (%)	Specificity (%)	Youden’s index
TyG Percentage Increase+BMI	0.071	0.684(0.631-0.736)	0.001	71.7	62.1	0.338
Early Pregnancy TyG+BMI	9.10	0.683(0.630-0.736)	0.001	75.3	82.1	0.574
Second Trimester TyG+BMI	9.85	0.731(0.684-0.779)	0.001	78.5	76.2	0.547

Bai et al. (37), based on a large-scale, multicenter maternal cohort study, found that an elevated TyG index in mid-pregnancy is not only closely associated with the onset of GDM but also significantly linked to an increased risk of various adverse pregnancy outcomes. It can significantly optimize the discriminatory power of traditional GDM risk prediction models, thereby corroborating the perspective of this study from the perspective of long-term prognosis: The mid-pregnancy TyG index accurately reflects the severity of overall maternal metabolic dysfunction and insulin resistance, serving as an excellent metabolic biomarker for both the development of GDM and comprehensive pregnancy management. A specialized screening study conducted by Sağnıç et al. (38). On pregnant women between 24 and 28 weeks of gestation further confirmed that the second trimester is the optimal time window for using the TyG index to identify high-risk populations for GDM. During this stage, TyG demonstrates stable and excellent diagnostic performance for GDM, once again illustrating that the use of second-trimester TyG for large-scale clinical prenatal GDM risk screening is simple, efficient, and highly scalable.

### 4.3 Percentage changes in TyG differences during early and mid-pregnancy and the risk of GDM, and their clinical implications

This study innovatively found that an increased percentage change in TyG from early to mid-pregnancy is an independent risk factor for GDM (adjusted HR = 2.564, 95% CI: 1.734, 3.790, P = 0.001), suggesting that a progressive increase in TyG during pregnancy better reflects the trajectory of metabolic deterioration and has greater predictive value than a single-point-in-time measure. Compared to static TyG, the percentage change in dynamic TyG values can avoid cross-sectional detection bias and more accurately reflect the rate of progression of insulin resistance.

Zhang (39) et al, based on a large-scale cohort study in Beijing, found that elevated TyG indices at all stages of pregnancy were associated with an increased risk of large-for-gestational-age (LGA) infants, and that TyG levels in the second trimester had significantly greater predictive value for adverse pregnancy outcomes than those in the first trimester. The study further confirmed through trajectory analysis that a sustained rise in TyG during pregnancy, or an increase from a low baseline, is an independent risk factor for fetal overgrowth, whereas a decrease in TyG from high to normal levels does not increase the risk of adverse outcomes. This conclusion is highly consistent with the present study, suggesting that the second trimester is a critical window during which the TyG index reflects metabolic disturbances and predicts GDM and adverse pregnancy outcomes, and that dynamic monitoring of TyG changes is more clinically meaningful than a single measurement. Additionally, this study provides important evidence-based support for the focus of our study on analyzing the mid-pregnancy TyG index and plotting ROC curves, confirming that mid-pregnancy TyG can serve as a stable, simple, and effective indicator for metabolic monitoring and risk screening during pregnancy.

All study participants were recruited from the Hunan Provincial Maternal and Child Health Hospital, making this a single-center study. Given the study’s geographical concentration in Hunan Province and the influence of regional dietary patterns, lifestyles, and ethnic characteristics, the generalizability of the findings may be limited; further validation through multicenter studies conducted in different regions across the country is required; Additionally, blood tests in this study were conducted using a single fasting measurement, which may introduce measurement errors. Furthermore, potential confounding factors such as dietary intake and physical activity levels were not included, which may influence the study conclusions. Future research could improve the analysis by incorporating repeated measurements and supplementing relevant data.

## 5 Conclusions

In summary, TyG in early pregnancy focuses on early warning, while the percentage increase in TyG difference emphasizes dynamic monitoring of trends in metabolic deterioration. In contrast, TyG in mid-pregnancy combines high-risk association strength, good predictive performance, and clinical feasibility. Together, these three approaches complement each other to form a comprehensive metabolic management system throughout pregnancy. Clinically, a monitoring protocol involving two TyG assessments—one in early pregnancy and one in mid-pregnancy—can be established. This approach allows for enhanced follow-up and intervention for patients with rapidly rising TyG levels, facilitating a shift from “single-point screening” to “comprehensive dynamic management” and provide a reference for early screening and management of GDM as well as the long-term prevention of metabolic diseases.In this study, all baseline blood samples were strictly collected within a narrow time window of 10–13^+4^ weeks of gestation; variations in individual gestational age were minimal, resulting in limited interference with TyG test results; therefore, gestational age at blood collection was not included in the analysis. This study did not systematically collect standardized data on dynamic weight gain during pregnancy; therefore, it is currently not possible to incorporate such data into the model for adjustment. Regarding potential confounding effects of dietary patterns, the lack of quantifiable data suitable for statistical analysis means that the conditions for adjustment have not yet been met; we will address this issue in future studies.

## Data Availability

The raw data supporting the conclusions of this article will be made available by the authors, without undue reservation.
